# Research on Carbonation Resistance of Modified/Non-Portland Cements in Carbon Capture, Utilization, and Storage-Enhanced Oil Recovery

**DOI:** 10.3390/ma19112279

**Published:** 2026-05-28

**Authors:** Yaqiong Cao, Rengguang Liu, Shiming Zhou, Qian Tao, Luo Liu

**Affiliations:** SINOPEC Research Institute of Petroleum Engineering Co., Ltd., 197 Baisha Road, Changping District, Beijing 102206, China; liurg.sripe@sinopec.com (R.L.); taoqian.sripe@sinopec.com (Q.T.); liul37309.sripe@sinopec.com (L.L.)

**Keywords:** CCUS-EOR, carbonation resistance, PANI@TiO_2_ composite, phosphoaluminate cement

## Abstract

**Highlights:**

- The carbonation resistance of three cement systems follows the order CAP > P@T > PT under simulated CCUS-EOR harsh environment.
- CAP appears to exhibit a potential “corrosion-induced densification” effect, with continuously improved compressive strength and reduced permeability after long-term CO_2_ corrosion.
- P@T modified cement suggests the decoupling of high carbonation degree and microstructural stability by regulating carbonate crystallization behavior.

**Abstract:**

Under the global carbon-neutrality target, the technology of carbon capture, utilization, and storage-enhanced oil recovery (CCUS-EOR) faces a severe challenge of carbonation-induced degradation of oil-well cement in harsh downhole environments. Traditional cement suffers serious structural failure under high-temperature and high-pressure CO_2_ conditions, whereas single-nanoparticle or polymer modification cannot meet long-term safety requirements. Meanwhile, the comparative study between the “matrix modification strategy” and the “cement system replacement strategy” is still insufficient under real CCUS-EOR conditions. In this study, experimental investigations including macroscopic performance testing, phase analysis, and multi-scale microstructural characterization were conducted. This study systematically evaluates the carbonation resistance of polyaniline@titanium dioxide-modified cement (P@T) and calcium aluminate phosphate cement (CAP). The results show that the carbonation resistance follows the descending order: CAP > P@T > silica-fume-containing Class G oil-well cement (PT). CAP seems to demonstrate a potential “corrosion-induced densification” effect. After 90 days of corrosion, its compressive strength increases to 62.5 MPa, and its permeability decreases to 13.3% of the initial value, indicating continuously improved performance. P@T indicates the possible decoupling of high carbonation degree (CaCO_3_ content of 25.26%) and microstructural stability through a structural regulation mechanism of “physical filling–homogeneous distribution of carbonation products”. In contrast, PT undergoes complete structural failure after 60 days. This study fills a gap in comparative evaluation between modification and replacement schemes, reveals the multi-scale structural regulatory effects of P@T and the intrinsic stability of CAP, and provides two reliable cement solutions—“modification enhancement” and “system replacement”—for CCUS-EOR environments. The scientific validity is demonstrated through multi-scale characterization, offering key theoretical and technical support for ensuring long-term wellbore integrity.

## 1. Introduction

Driven by the global carbon neutrality goal, CCUS-EOR technology has become the core pathway for emission reduction and energy efficiency improvement [1]. As the key barrier for interlayer isolation and wellbore stability, oil-well cement directly determines the safe operation of CCUS-EOR projects. Its carbonation degradation in harsh CO_2_-rich environments has become a critical technical bottleneck restricting the large-scale application of this technology [2].

Class G oil-well cement is a typical basic oil-well cement specified in API Spec 10A standard, which is widely used in conventional oil–gas well and CCUS well cementing engineering due to its stable performance and good adaptability to high-temperature environments [3]. However, pure Class G cement exhibits poor carbonation resistance in harsh CCUS-EOR environments. High CO_2_ partial pressure accelerates the diffusion of corrosive media and the consumption of alkaline products [4].

To address this issue, researchers have proposed various modification strategies: Mineral admixtures (e.g., silica fume) can improve high-temperature stability through pozzolanic reactions but fail to fundamentally inhibit CO_2_ diffusion and alkali consumption [5]; nanoparticles (e.g., TiO_2_) can fill nanopores and promote hydration, but they are prone to agglomeration under high-temperature and high-shear downhole conditions, resulting in weakened interface bonding and failed long-term protection [6,7,8]; conductive polymers (e.g., polyaniline, PANI) have attracted significant attention in carbonation resistance modification due to their proton buffering capacity and electrochemical activity [9]. Meanwhile, conductive polymers can provide proton buffering, interfacial adhesion, and electrostatic repulsion to inhibit acid corrosion and decalcification [10]; PANI in particular can form a protective layer on the hydration product surface and slow down carbonation kinetics. However, single PANI lacks sufficient physical barrier effect, and its stability is insufficient under high-pressure CO_2_ corrosion for a long time [10].

Therefore, the PANI@TiO_2_ composite combines the comprehensive properties of polyaniline and titanium dioxide, which is expected to overcome the defects of single modification under the harsh conditions of 130 °C/25 MPa. However, the application effect and multi-scale reinforcement mechanism of PANI@TiO_2_ composites (prepared by combining PANI with TiO_2_) under harsh CCUS-EOR conditions remain unclear.

Meanwhile, the development of alternative cement systems with intrinsic carbonation resistance has become a research hotspot [11]. Calcium aluminate phosphate cement, as a typical low-calcium non-Portland cement, has gradually attracted attention for CO_2_ storage wells due to its low calcium content, low alkalinity, and inherent acid resistance [12,13]. Phosphoaluminate cement (CAP) forms hydration products mainly composed of calcium alumino-phosphate hydrates and calcium phosphate hydrates, hardly producing Ca(OH)_2_, which is prone to reacting with CO_2_. Thus, it exhibits high chemical stability in acidic environments [14]. However, most existing studies focus on ambient or low-pressure conditions [15]. Nevertheless, in-depth studies on the carbonation behavior, long-term performance of CAP in CCUS-EOR environments, and its systematic performance comparison with modified cement are still lacking. Moreover, few studies have simultaneously evaluated “matrix modification” and “cement system replacement” to clarify which strategy is more reliable for long-term wellbore integrity under harsh CCUS-EOR conditions.

To ensure comparability between the two technical routes, the dosages of 1 wt% PANI@TiO_2_ and 5 wt% sodium hexametaphosphate were selected as the optimal dosages based on theoretical analysis and preliminary pre-experiments: 1 wt% PANI@TiO_2_ can achieve sufficient interface modification without agglomeration [16,17], and 5 wt% sodium hexametaphosphate can balance the rheology and hydration rate of CAP slurry [18,19].

Therefore, to fill the abovementioned research gaps, this study aims to evaluate the carbonation resistance of PANI@TiO_2_-modified cement and phosphoaluminate cement under simulated CCUS-EOR conditions (130 °C, 25 MPa, CO_2_ partial pressure of 7 MPa) and to explore their carbonation resistance mechanisms. The findings are expected to provide theoretical guidance and technical support for the development of high-performance oil-well cement materials suitable for CCUS-EOR environments and to enrich understanding of the carbonation mechanisms of cementitious materials in harsh CO_2_-rich environments.

## 2. Materials and Methods

### 2.1. Materials

The chemical compositions of Class G oil-well cement (Sichuan Jiahua Special Cement Co., Ltd., Leshan, China) and CA50-grade aluminate cement (Zhengzhou Jianai Special Aluminate Cement Co., Ltd., Zhengzhou, China) are listed in Table 1. Silica fume (300 mesh), organic silicon defoamer, polycarboxylate superplasticizer, retarder (Dezhou Continental Shelf Petroleum Engineering Technology Co., Ltd., Dezhou, China), and sodium hexametaphosphate (Hubei Xingfa Chemical Group Co., Ltd., Jinzhou, China) are all industrial-grade additives commonly used in cementing, with clear functions and stable performance.

The PANI@TiO_2_ composite was prepared by in situ polymerization. Aniline (1 mL) was dispersed in 1 mol/L HCl solution (150 mL), and TiO_2_ (1.75 g, n(PANI):n(TiO_2_) = 1:2) was added. The mixture was stirred in an ice-water bath for 30 min, then mixed with ammonium persulfate (2.40 g, dissolved in 50 mL 1 mol/L HCl) for a reaction for 4 h. After washing and drying, a dark blue powder with a particle size of 0.3~0.5 μm was obtained. Materials used for the preparation of PANI@TiO_2_ composite were purchased from Shanghai Aladdin Biochemical Technology Co., Ltd., Shanghai, China.

### 2.2. Preparation and Corrosion of Cement Samples

#### 2.2.1. Preparation of Cement Samples

The proportions of the mixed cement systems (PT, P@T) and the CAP system are shown in Table 2. The mix proportion was optimized based on the following considerations: A silica fume content of 35% balances high-temperature stability and alkali content control [5]; a PANI@TiO_2_ content of 1% was determined based on preliminary pre-experiments (balancing modification effect and economy); a sodium hexametaphosphate content of 5% in the CAP system was used to regulate the slurry rheology. Cement slurry was stirred at 12,000 rpm for 35 s using a constant-speed mixer (TG-3060 A, Shenyang Taige Petroleum Instrument Equipment Co., Ltd., Shenyang, China), which is consistent with the API standard (API RP 10B-2-2013 (R2019)) [20] and high-shear mixing for laboratory evaluation of oil-well cement. Then, they were poured into 50.8 × 50.8 × 50.8 mm^3^ molds (complying with API standards). After curing at 130 °C and 25 MPa for 7 days, cylindrical samples of Φ25 mm × 50 mm were drilled. This size design ensures uniform penetration of corrosive media and meets the requirements of subsequent tests.

#### 2.2.2. Corrosion of Cement Samples

Corrosion experiments were conducted in a high-temperature and high-pressure autoclave (TL-3, Jingzhou Taling Machinery Co., Ltd., Jingzhou, China). The composition of simulated water refers to the actual formation water salinity of the million-ton CCUS well in Shengli Oilfield (Table 3). The ion concentrations were determined by inductively coupled plasma optical emission spectrometry (ICP-OES, Agilent 720ES, Agilent Technologies, Santa Clara, CA, USA) and ion chromatography (ICS-600, Thermo Fisher Scientific, Waltham, MA, USA), with measurement error controlled within ±2%. The fluid-to-cement volume ratio in the autoclave was fixed at 10:1 to maintain constant hydrodynamic conditions and a consistent carbonation rate across all parallel experiments, ensuring reproducibility of test results. The corrosion conditions were set to 130 °C and 25 MPa (CO_2_ partial pressure of 7 MPa). Temperature and pressure were monitored in real time during corrosion, with errors controlled to ±0.5 °C and ±0.1 MPa to ensure the stability of test conditions.

### 2.3. Experimental Methods

#### 2.3.1. Phenolphthalein Method

The phenolphthalein coloration method (1% phenolphthalein ethanol solution) was used to determine the carbonation depth by measuring the color boundary distance in the cross-section. For Portland-based systems (PT, P@T), this depth approximates carbonation depth; for CAP, it reflects the alkalinity loss front rather than true carbonation depth. This method is intuitive and widely used in cement carbonation evaluation [21], with a test error of ≤0.1 mm.

#### 2.3.2. Compressive Strength

Compressive strength was tested using a universal mechanical testing machine (HY-20080, Shanghai Hengyi Precision Instrument Co., Ltd., Shanghai, China) at a loading rate of 1.2 kN/s. Three parallel samples were tested for each group, and the average value was taken. This loading rate complies with the GB/T 17671-2021 standard [22], which can accurately reflect the bearing capacity of cement stone. Three parallel specimens from the same batch were tested for each aging time; separate specimens were used for each exposure duration.

#### 2.3.3. Permeability and Porosity

After drying the samples at 60 °C for 24 h, permeability and porosity were tested using a core flow tester (LDY 50–180, Nantong Yichuang Experimental Instrument Co., Ltd., Nantong, China). Drying treatment prevents moisture interference in permeability testing and ensures data accuracy. Permeability was measured under the steady-state gas-flow method with nitrogen as the test medium, at a confining pressure of 3 MPa and an inlet pressure of 0.5 MPa. Porosity was determined by the gas expansion method based on Boyle’s law. Separate specimens were used for each aging time.

#### 2.3.4. Statistical Analysis and Property Change Rate Calculation

To quantitatively characterize the performance evolution of cement specimens under CO_2_ corrosion, the compressive strength change rate, permeability variation rate, and porosity variation rate were calculated based on the tested data. Statistical comparisons of compressive strength, permeability, and porosity among the three groups at each corrosion age were performed using one-way analysis of variance (ANOVA) followed by Tukey’s post hoc multiple comparison test. The confidence level was set at 95%, corresponding to a significance level of α = 0.05. Differences with *p* < 0.05 were considered statistically significant, which were indicated by different lowercase letters above the bars in the corresponding figures. All tests were carried out on three parallel specimens, and the results were reported as the mean value.

The calculation formula of the property change rate is as follows:

Change rate (%) = [(Test value at corrosion age − Initial value)/Initial value] × 100%

#### 2.3.5. Phase Composition Analysis

X-ray diffraction (XRD, Ultima IV, Malvernpanalytical, Malvern, Worcestershire, UK) (scanning speed of 0.08°/s, 2θ = 5°~70°) was used for qualitative analysis of phase composition, and a thermal analyzer (TG, TG-DTA8122, Rigaku, Akishima, Tokyo, Japan) (heating rate of 20 °C/min) was used for quantitative determination of CaCO_3_ content. The thermal analysis was conducted at a heating rate of 20 °C/min from room temperature to 900 °C under a N_2_ atmosphere; the CaCO_3_ content was calculated from the mass loss in the range 600–800 °C corresponding to calcite decomposition, using separate specimens for each aging time. The combination of these two methods can comprehensively reveal the phase evolution law.

#### 2.3.6. Microstructural Analysis

X-ray computed tomography (XCT, Phoenix V tome × M300, Baker Hughes, Houston, TX, USA) (parameters shown in Table 4), a high-precision industrial CT with a large field of view suitable for cement core samples, was used for non-destructive characterization of three-dimensional defect evolution. Defect segmentation was performed using Otsu’s thresholding method to separate the solid matrix from pores and cracks. And scanning electron microscopy–energy dispersive spectroscopy (SEM-EDS; SU5000, Hitachi, Chiyoda-ku, Tokyo, Japan) was used to analyze the cross-sectional morphology and elemental distribution. Separate specimens were used for XCT and SEM-EDS at each aging time. For SEM testing, samples were cut into small pieces and dried at 60 °C for 24 h. For backscattered electron (BSE) imaging of cement specimens, the dried samples were vacuum-impregnated with epoxy resin, ground, and polished to a flat surface. Samples were sprayed with gold–palladium alloy under vacuum for 120 s to improve conductivity. This combined characterization can analyze structural changes from macro to micro scales and from overall to local scales, providing direct evidence for mechanism analysis.

## 3. Results

### 3.1. Evolution of Macroscopic Performance

#### 3.1.1. Evolution of Macroscopic Morphology and Corrosion Depth

**Figure 1:**
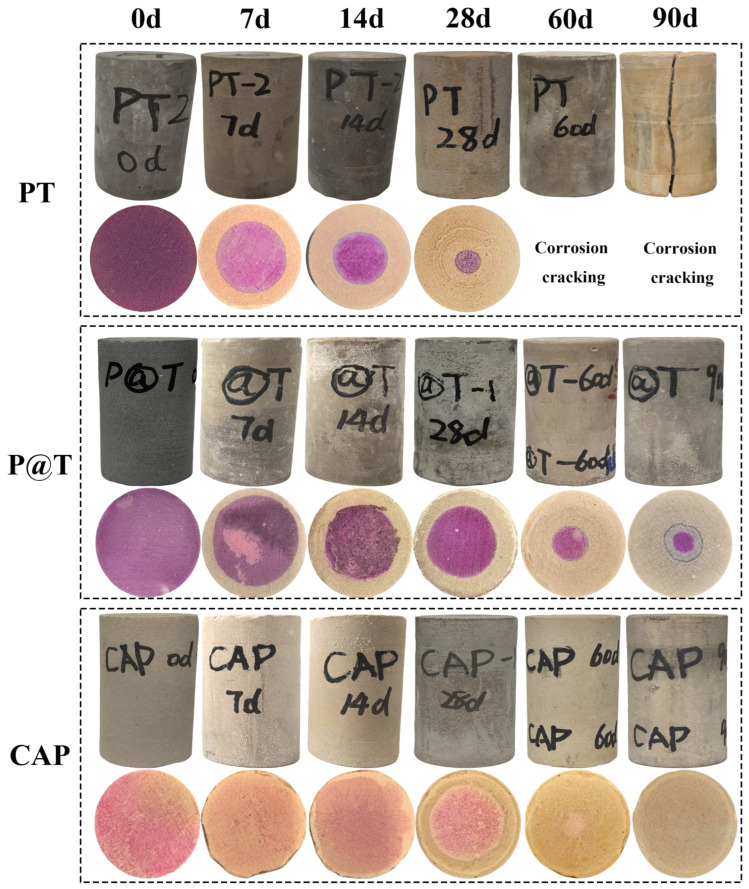
Macroscopic morphologies and corrosion depths of cement stones in different systems at various corrosion ages.

Macroscopic morphology observation and phenolphthalein coloration depth analysis (Figure 1) collectively revealed distinct damage forms and carbonation processes in the three groups of materials under CO_2_ corrosion, intuitively reflecting the differences in their carbonation resistance.

In terms of macroscopic morphology evolution, the PT group exhibited a progression from surface yellowing to obvious cracking at 60 days, with complete loss of structural integrity. Although the surface color of the P@T group changed, no cracks appeared throughout the process, and the structure remained intact. The CAP group exhibited optimal stability, with only mild color changes and no macroscopic damage throughout the entire process.

Regarding carbonation depth and mode, cross-sectional analysis by phenolphthalein coloration provided a microscopic explanation. The carbonation reaction of the PT group was the most intense, and the carbonation layer propagated rapidly inward. By 28 days, the uncarbonated core had significantly shrunk, and it could no longer be observed due to severe corrosion and cracking. The carbonation process of the P@T group was effectively regulated: the carbonation layer propagated slowly inward from the edge for 7 days, and a distinct uncarbonated area remained in the core even at 90 days, indicating that the modifier significantly delayed the penetration and reaction of CO_2_. The carbonation mode of the CAP group was completely different: due to its low initial alkalinity, the cross-sectional color transitioned uniformly from pink to light brown. Although almost the entire cross-section showed color change at 90 days, this does not indicate severe carbonation, as the structure remained dense and intact throughout the process, consistent with low CaCO_3_ formation and preserved microstructure.

#### 3.1.2. Evolution Law of Compressive Strength

**Figure 2:**
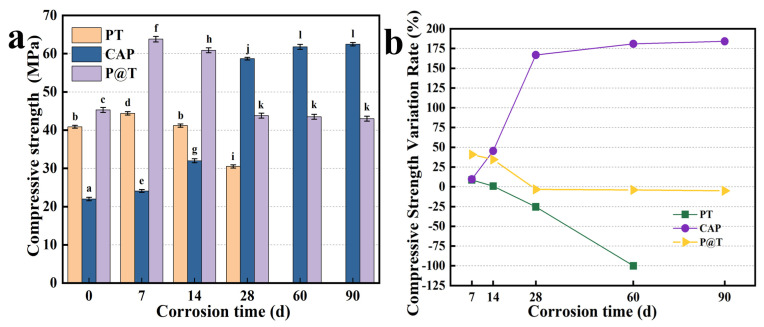
Compressive strength (**a**) and compressive strength change rate (**b**) of different systems at different corrosion ages.

The evolution law of compressive strength (Figure 2) revealed the characteristics of “general initial enhancement and significant long-term differentiation” of the three cement systems under CO_2_ corrosion. Statistical analysis using one-way ANOVA followed by Tukey’s multiple-comparison test (*p* < 0.05) confirmed that, at each corrosion age, the compressive strengths of the three systems exhibited significant pairwise differences. As indicated by different lowercase letters above the bars in Figure 2a (at the same corrosion age, different letters denote statistically significant differences among groups), this statistical validation supports the observed reliability of performance differentiation under CO_2_ corrosion.

At the early corrosion stage (0–7 days), all three systems showed strength gain. On day 0, the P@T system already displayed the highest strength (45.3 MPa, marked as letter c), significantly outperforming the PT system (40.9 MPa, b) and the CAP system (22.0 MPa, a), with all three groups differing significantly from each other. By day 7, the P@T system achieved the greatest strength increase, reaching a peak of 63.8 MPa (f). This enhancement is attributed to the heterogeneous nucleation effect of PANI@TiO_2_ [23], which refines grain size and optimizes pore structure [16], belonging to the combined effect of chemical hydration promotion and physical microstructure refinement [16]. The PT system reached 44.4 MPa (d), exhibiting a moderate increase, while the CAP system only increased to 24.1 MPa (e), remaining the lowest among the three.

From day 7 to day 14, the trends in strength evolution began to diverge clearly. The P@T system maintained a relatively high strength level (60.9 MPa, h), although it decreased slightly from the peak at day 7. Meanwhile, the CAP system accelerated its strength development, reaching 32.0 MPa (g), narrowing the gap with the PT system (41.2 MPa, b). Notably, the Tukey comparison results confirmed that the PT system showed no significant difference in strength between day 0 and day 14.

At the middle-to-late corrosion stage (14–28 days), the performance differences became more pronounced. The strength of the CAP system rose sharply to 58.7 MPa (j), surpassing that of the P@T system (43.8 MPa, k) and the PT system (30.5 MPa, i). This strength reversal marks the onset of its “corrosion-induced densification” effect, whereby carbonation products fill the matrix pores and increase compactness. At the same time, the strength of the P@T system continued to decline slowly, whereas the PT system entered a phase of continuous strength deterioration. Tukey’s test further revealed that the strength of the CAP system at day 28 was significantly higher than its values at earlier ages, confirming the steadily increasing densification effect.

At the late corrosion stage (60–90 days), the three systems exhibited markedly different long-term corrosion stability. The CAP system showed exceptional durability, with its strength increasing to approximately 62 MPa (l) at both 60 and 90 days, representing a ~184% increase relative to its initial strength (Figure 2b). This sustained strength enhancement confirms the dominant role of the corrosion-induced densification mechanism, whereby ongoing carbonation reactions produce stable calcium carbonate phases that progressively densify the microstructure over time. The Tukey results also showed that the strength of the CAP system at 60 and 90 days did not differ significantly, indicating that a stable protective layer had formed to resist further corrosion. The strength of the P@T system stabilized at approximately 43 MPa (k) at 60 and 90 days, but by this time, it was inferior to that of the CAP system. The strength change rate curve (Figure 2b) shows that its early strengthening effect gradually diminished, with the curve flattening after day 28. Combined with the Tukey comparisons, this confirms that the PANI@TiO_2_ modification effectively slowed long-term strength degradation but did not completely prevent it, as its strength remained stable yet could not match the progressive densification of the CAP system. All PT specimens suffered severe corrosion cracking and structural failure at 60 and 90 days, resulting in no valid compressive strength data at these two ages, underscoring the poor long-term resistance of the PT system to CO_2_ corrosion.

It is worth noting that the initial enhancement amplitude was not positively correlated with long-term corrosion resistance. Although the P@T group had the largest initial enhancement amplitude, its long-term performance was still inferior to that of the CAP group, which continued to strengthen. This phenomenon indicates that chemical stability and the mechanism of microstructural evolution within the material system itself, rather than the short-term enhancement effect, are the key factors determining its long-term durability in harsh CO_2_ environments, which is consistent with the existing understanding that “short-term performance cannot reliably characterize long-term durability”.

#### 3.1.3. Coupled Evolution of Permeability and Porosity

**Figure 3:**
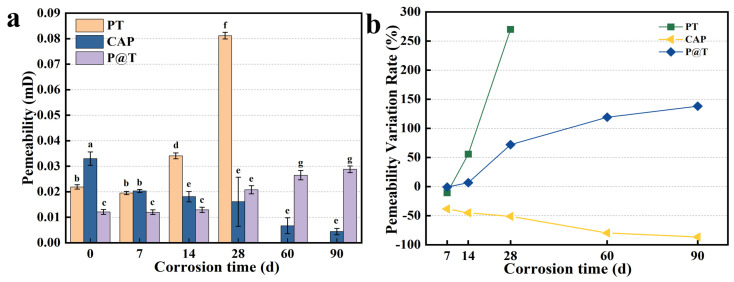
Permeability (**a**) and permeability variation rate (**b**) of different systems at different corrosion ages.

**Figure 4:**
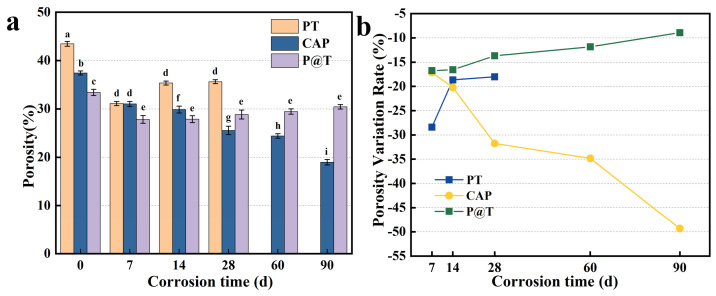
Porosity (**a**) and porosity variation rate (**b**) of different systems at different corrosion ages.

The synchronous evolution of permeability (Figure 3) and porosity (Figure 4) reveals, from the perspectives of mass transport and structural integrity, the fundamental differences among the three cement systems under CO_2_ corrosion. Their variation patterns are highly coupled with the macroscopic compressive strength, constituting key microscopic criteria for distinguishing long-term durability. One-way ANOVA followed by Tukey’s multiple comparison test (*p* < 0.05, indicated by different lowercase letters above the bars in Figure 3a and Figure 4a) confirmed that, at most corrosion ages, significant differences in both permeability and porosity exist among the three groups.

The CAP group exhibits the best structural densification: Its porosity continuously decreases from 37.4% to 18.97%, and its permeability drops to 0.0044 mD at 90 days (only 13.3% of its initial value). On day 0, CAP has the highest permeability (0.032 mD, a) and the second-highest porosity (37.4%, b), both significantly different from PT (0.021 mD, b; 43.5%, a) and P@T (0.012 mD, c; 33.2%, c). As corrosion proceeds, by day 28, the permeability of CAP decreases to 0.016 mD (e) and porosity to 25.5% (g), outperforming PT (0.0812 mD, f; 35.6%, d) and P@T (0.020 mD, e; 28.1%, e). At 60–90 days, the permeability of CAP stabilizes at 0.004–0.006 mD (e) and porosity at 18.97–24.3% (i/h), significantly lower than those of the late-stage P@T group (0.026–0.0288 mD, g; 29.0–30.4%, e), which is also reflected in the negative variation rates in Figure 3b and Figure 4b. The Tukey results verify the significant differences in permeability and porosity of CAP between early and late corrosion ages, consistent with its progressive densification mechanism.

The P@T group exhibits stable and gentle fluctuations: Porosity varies slightly between 27.8% and 30.4%, and permeability increases only slowly to 0.0288 mD. On day 0, P@T has the lowest permeability (0.012 mD, c) and porosity (33.2%, c), which are significantly lower than those of both CAP and PT. Throughout the corrosion period, its permeability and porosity remain consistently lower than those of the early-stage CAP and mid-stage PT (e.g., c vs. a/b at 0 d; e vs. f at 28 d). By 60–90 days, the permeability rises to approximately 0.028 mD (g) and porosity to approximately 30% (e), which are significantly higher than those of the late-stage CAP group but far lower than the peak values of the PT group. The slow and controlled increase in the variation rates (Figure 3b and Figure 4b) confirms that the modification retards structural degradation but does not fully prevent it, consistent with its strength evolution trend.

In contrast, the PT group undergoes a degradation process of initial shrinkage followed by rapid expansion: Porosity rebounds to 35.6% at the middle stage, and its permeability surges by 269.9% to 0.0812 mD within 28 days, indicating the formation of a connected pore network and the loss of structural load-bearing capacity. On day 0, PT has intermediate permeability (0.021 mD, b) and the highest porosity (43.5%, a), significantly different from both CAP and P@T. By day 28, its permeability reaches 0.0812 mD (f) and porosity rebounds to 35.6% (d), both significantly higher than those of CAP (e/g) and P@T (e). This sharp jump, combined with the steep increase in the variation rates (Figure 3b and Figure 4b), indicates severe pore coarsening and crack formation, consistent with its later macroscopic structural failure. The Tukey test further confirms that the permeability and porosity of PT at day 28 are significantly higher than those at all earlier ages, highlighting irreversible structural damage.

These microstructural differences directly regulated and explained the differentiation of macroscopic performance. The continuous densification of the CAP group not only constructed an effective penetration barrier for corrosive media but also provided structural support for its compressive strength to continuously increase to 62.5 MPa; the malignant expansion of pores in the PT group directly led to a sharp increase in permeability and strength degradation until destruction; the gentle evolution of the P@T structure was consistent with its strength trend of “first peak and then slow decline”. 

### 3.2. Phase Evolutions

#### 3.2.1. XRD Phase Analysis

**Figure 5:**
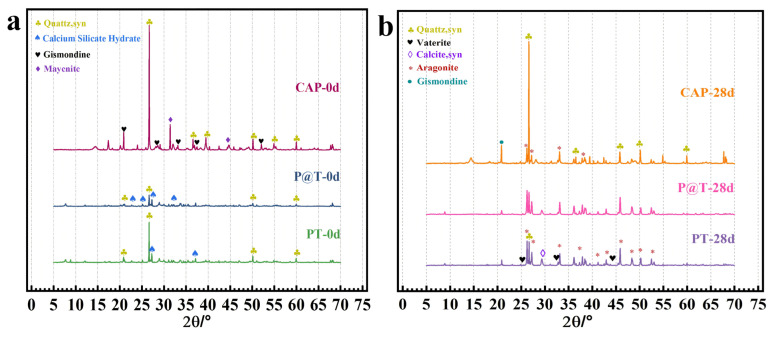
XRD patterns of cement stones in different systems at different corrosion ages. (**a**), the corrosion age of 0 days; (**b**), the corrosion age of 28 days.

XRD phase analysis (Figure 5) clearly revealed that the CAP group followed a fundamentally different response path from the PT and P@T groups under CO_2_ corrosion, which directly determined the difference in their macroscopic durability.

After 28 days of corrosion, the phase evolution of the two systems was completely different. The PT and P@T systems underwent complete carbonation: The diffraction peaks from various calcium carbonate crystals, such as calcite, aragonite, and vaterite, appeared. In sharp contrast, the CAP system underwent only a selective phase transformation: the diffraction peak of its characteristic phase ettringite disappeared, and a new aragonite phase formed, while the structural skeletons such as quartz and gismondine were retained, and the corrosion process was mild and controlled.

The above path difference is rooted in the inherent differences in their initial chemical compositions. Thanks to its low calcium content, the CAP system hardly generates C–H that is prone to reacting with CO_2_, significantly reducing the carbonation driving force and reaction intensity at the source of reactants. This mechanism is consistent with the conclusions of existing studies [14], and this study further confirms the effectiveness of this characteristic in the harsh environment of simulated CCUS-EOR with high temperature and high CO_2_ partial pressure. In contrast, the cement system is rich in C–S–H and C–H, which are prone to carbonation, providing an inherent driving force for its complete carbonation decomposition.

It is worth noting that the XRD patterns of the P@T system and the PT system were highly consistent before and after corrosion. This confirms that the PANI@TiO_2_ composite did not change the composition of hydration products of the cement matrix and the basic carbonation reaction essence in a CO_2_ environment. The main role of the modifier is to delay rather than block this fundamental degradation process through physical barriers and interface regulation.

Therefore, phase analysis shows that fundamentally improving the long-term durability of materials in harsh CO_2_ environments requires changing the chemical nature of the cementitious material system, which has greater potential than physicochemical modification in the existing system.

#### 3.2.2. Quantitative Thermogravimetric Analysis

**Figure 6:**
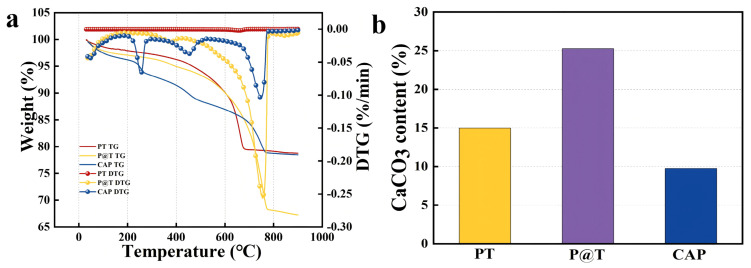
DTG curves (**a**) and CaCO_3_ content changes (**b**) in cement stones in different systems at 28 days of corrosion.

Thermogravimetric analysis (Figure 6) quantitatively revealed significant differences in the content of carbonate (CaCO_3_) products of the three types of cement after 28 days of corrosion: the P@T group was the highest (25.26%), followed by the PT group (14.98%), and the CAP group was the lowest (9.77%). This order cannot be directly equated with the degree of carbonation damage but reveals three distinct coupled mechanisms of “carbonation reaction-structural evolution”.

The low content in the CAP group is due to its intrinsic chemical stability. Its low-calcium characteristics prevent the formation of a large number of carbonation-prone phases, inhibiting violent carbonation reactions at the source; thus, the total amount of carbonates generated is limited, which is consistent with existing understanding. The relatively low content of the PT group is precisely the consequence of its rapid structural collapse. Its early loose pore structure accelerates the transport and reaction of corrosive media, leading to premature matrix damage. Some of the generated carbonates cannot be stably retained and are leached by seepage before, so the measured cumulative amount is relatively low. The high content of the P@T group is a key finding of this study. Its high CaCO_3_ content did not cause structural degradation like the PT group. The key lies in the fact that the PANI@TiO_2_ composite modifier regulated the crystallization behavior of carbonates. In addition, the possibility of CO_2_ physical adsorption/trapping at the PANI interface cannot be excluded [24,25]. The -NH- functional groups in PANI have a certain interaction with CO_2_, which may temporarily retain some CO_2_ at the interface and reduce its direct reaction with C–S–H in the early stage. However, long-term results show that most carbonates still come from the carbonation reaction of hydration products, and the core mechanism is the regulation of carbonate crystallization behavior. It promoted the generated CaCO_3_ particles to refine and distribute uniformly, thereby filling pores and optimizing the structure, rather than causing product accumulation, expansion, and cracking. This implies the possible decoupling of “high carbonation degree” and “microstructural stability”, breaking the cognitive framework in traditional research that “high carbonation is inevitably accompanied by structural degradation” [26], which is one of the core innovations of this study.

The intensity of the decomposition peak at 600–800 °C in the DTG curve (P@T > PT > CAP) is completely consistent with this quantitative result, cross-validating the above conclusions. Therefore, when evaluating the durability of cement in CO_2_ environments, the CaCO_3_ content must be interpreted in combination with microstructural evolution: high accumulation may result from controlled, benign reactions, while low accumulation may indicate complete structural destruction or intrinsic low reactivity.

## 4. Discussion

### 4.1. Multi-Scale Structural Characterization

To systematically clarify the damage mechanisms of different cement systems under CO_2_ corrosion, scanning electron microscopy (SEM) and X-ray computed tomography (XCT) were comprehensively used to reveal their structural evolution laws from multiple scales, such as two-dimensional microscopic morphology, elemental distribution, and three-dimensional spatial structure.

#### 4.1.1. Evolution of Three-Dimensional Defect Structure

**Figure 7:**
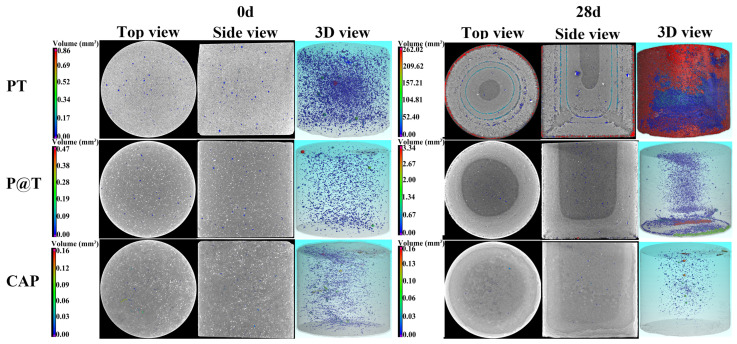
2D and 3D images of cement stones in different systems at different corrosion ages.

XCT three-dimensional imaging quantitatively revealed three distinct structural evolution paths (Figure 7). Before corrosion, the CAP group had the optimal initial compactness with a defect volume ratio of only 0.14% (Table 5); after physical filling optimization, the defect ratio of the P@T group was 0.19%, while that of the PT group was 0.50%. Each group was tested with three parallel samples, and the average value was used. After 28 days of corrosion, the evolution showed fundamental differentiation. The CAP group is inferred to show a potential “corrosion densification” effect, with the defect volume ratio decreasing counterintuitively to 0.07%, attributed to the secondary filling of pores by corrosion products such as aragonite [27]. Although aragonite filling reduces the defect rate, long-term durability beyond 90 days needs further attention. Sustained carbonate generation may introduce local internal stress or micro-cracking in ultra-long service periods, which requires long-term corrosion evaluation of more than 180 days in future research; the structure of the P@T group remained stable, with the defect ratio only slightly increasing to 0.31%, indicating that the modified layer effectively inhibited defect expansion; the PT group suffered structural collapse, with the defect ratio surging to 5.21%, and a large number of connected pore-crack networks were visible in the images.

#### 4.1.2. Microscopic Morphology and Composition Uniformity

**Figure 8:**
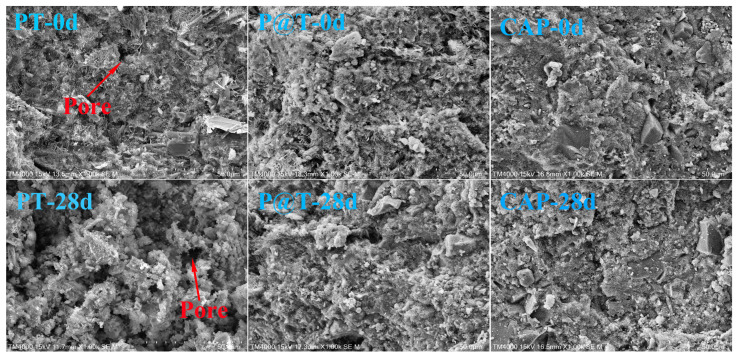
Cross-sectional SEM images of cement stones in different systems at different corrosion ages.

**Figure 9:**
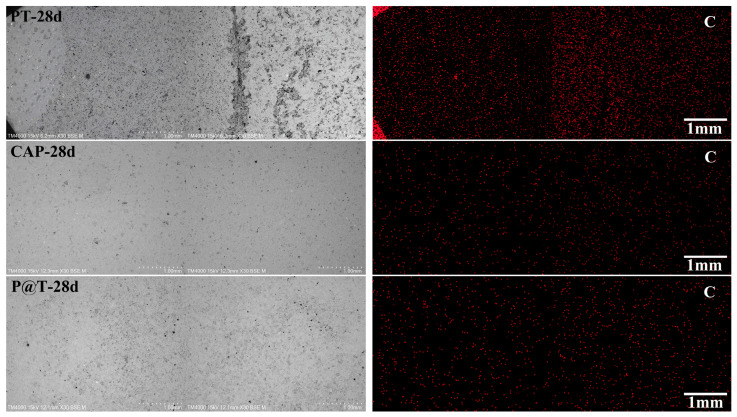
Backscattered images and C element distribution of the cross-section of cement stone in different systems at 28 d corrosion age.

SEM analysis (Figure 8) provided microscopic evidence for the above three-dimensional evolution. After corrosion, the cross-section of the PT group showed severe degradation, with interconnected pores and loose products; the morphology of the P@T group remained relatively intact; the CAP group was even denser. Backscattered electron (BSE) imaging (Figure 9) further revealed from the compositional level that the BSE image of the PT group showed gray disorder and stratification, indicating that its elemental distribution was uneven due to corrosion; the BSE images of the CAP group and the P@T group had uniform gray levels, confirming that the integrity of their structural composition was maintained.

#### 4.1.3. Decoupling of Carbonation Product Behavior and Structural Stability

C element mapping (Figure 9) and thermogravimetric analysis data collectively clarified the key role of carbonation product behavior. The C element distribution of the PT group was spatially heterogeneous (enriched in the carbonated layer and sparse in the leached layer), which was consistent with its relatively high CaCO_3_ content (14.98%) and accompanying structural damage. The C element signal of the CAP group was sparsely distributed, corresponding to its lowest CaCO_3_ content (9.77%) and intrinsic low reactivity. Notably, although the P@T group had the highest CaCO_3_ content (25.26%), its C element distribution was uniform, and the structure did not deteriorate. This indicates that PANI@TiO_2_ modification does not inhibit carbonation but suggests the potential decoupling of “high carbonation degree” and “structural stability” by regulating the crystallization behavior (refinement and uniform precipitation) of carbonate products. This conclusion is consistent with the regulation mechanism of nanocomposites on carbonate crystallization reported in previous studies [28,29] and further extends it to high-temperature and high-pressure CCUS-EOR environments.

In summary, multi-scale characterization clearly outlines the failure and reinforcement mechanisms of the three systems: the PT system undergoes cascading degradation from micro to three-dimensional due to product decomposition; the P@T system delays and regulates the degradation process through interface modification; the CAP system achieves structural self-densification by virtue of stable corrosion products. This provides direct structural evidence for understanding the differences in their macroscopic performance.

The laboratory cylindrical sample (Φ25 mm × 50 mm) is a standard size for evaluating cement corrosion resistance, which can reflect the intrinsic performance of the material. In the field wellbore, the material’s anti-carbonation mechanism remains consistent: the barrier effect of P@T and the self-densification of CAP will still play a dominant role. The small sample size accelerates the corrosion process, which can predict the long-term performance of the field-scale cement sheath in a short time.

### 4.2. Carbonation Resistance Mechanisms of Different Cement Systems

Based on the results of macroscopic performance and multi-scale characterization, the fundamental reason for differences in carbonation resistance among the three cement systems lies in their inherent differences in reinforcement mechanisms. The P@T system and the CAP system represent two effective strategies: “extrinsic active protection” and “intrinsic stability”, respectively.

#### 4.2.1. Multi-Scale Synergistic Active Protection System

**Figure 10:**
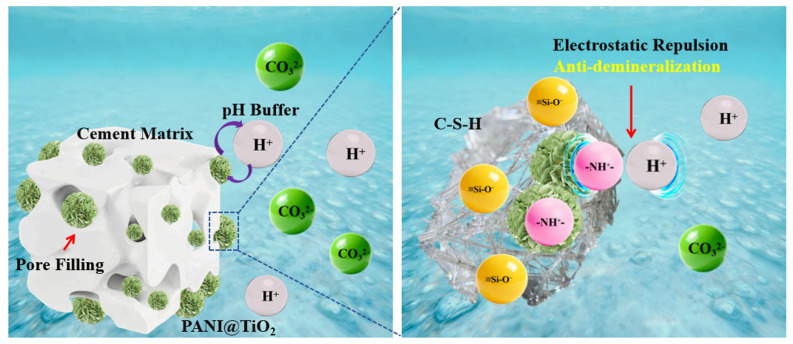
Schematic diagram of the carbonation resistance enhancement mechanism of PANI@TiO_2_ modified cement stone.

The PANI@TiO_2_ composite may impart a multi-scale active protection mechanism that potentially transcends conventional physical barrier effects (Figure 10). Recent studies on organic–inorganic nanocomposites in cement science have suggested that nanoscale fillers coupled with conductive polymers can synergistically enhance carbonation resistance through pore refinement, interfacial regulation, and proton buffering [16,30,31]. The PANI@TiO_2_ composite developed herein is presumed to follow similar principles but may exhibit superior performance under harsh CCUS-EOR conditions (130 °C, 25 MPa). At the nanoscale, TiO_2_ nanoparticles may fill the inter-C–S–H nanopores and reduce media diffusivity; at the molecular level, PANI may provide proton buffering and electrostatic repulsion, thereby delaying the kinetics of acidification and decalcification [10,32,33].

Numerical benchmarking against reported CCUS cement systems further highlights the effectiveness of the P@T system. Under comparable high-temperature and high-pressure CO_2_ environments, advanced geopolymers have been reported to achieve a 28-day compressive strength up to 48 MPa and permeabilities of 0.05–0.20 mD [34,35]; carbon-nanotube or graphene-reinforced cement composites are documented to exhibit compressive strengths of 45–58 MPa and permeabilities of 0.04–0.15 mD [30,36]. In contrast, the P@T system maintains a permeability of only 0.0288 mD at 90 d while retaining a compressive strength above 43 MPa (>43 MPa), outperforming most reported nanocomposite and geopolymer systems in terms of transport resistance and long-term structural stability. Notably, although the compressive strength of the P@T system is slightly lower than the peak values of some geopolymers (e.g., 48 MPa at 28 d [35]), its substantially lower permeability (0.0288 mD vs. ≥0.04 mD for the best-performing nanocomposites) underscores its superior resistance to CO_2_ transport under harsh CCUS-EOR conditions.

Multi-scale characterization (XCT/SEM) further differentiates the P@T system from conventional modified cements. Most nanomodified systems may reduce porosity but could still exhibit localized pore coarsening and heterogeneous carbonation product distribution under long-term CO_2_ exposure [30,37]. In contrast, the P@T system appears to show uniform carbonate precipitation and minimal pore coarsening, which could directly support a unique decoupling effect between high carbonation degree (25.26% CaCO_3_) and structural stability. This mechanism—carbonate refinement and homogeneous distribution potentially regulated by PANI@TiO_2_—may represent a novel contribution to the field, as it might challenge the conventional paradigm that high carbonation inevitably leads to structural degradation [38].

#### 4.2.2. Intrinsic Corrosion Resistance Path with Endogenous Stability

The calcium aluminate phosphate (CAP) cement exhibits exceptional long-term durability driven by its intrinsic low-calcium chemistry and corrosion-induced densification. Benchmarking against leading CCUS-resistant binders, geopolymers, and high-alumina cements typically reach 90 d strengths of 50–58 MPa under CO_2_-rich conditions [39,40]. In comparison, the CAP system achieves 62.5 MPa at 90 d, representing an approximately 8–25% strength advantage, while its permeability decreases to only 0.0044 mD—far lower than most reported high-performance systems (>0.04 mD) [41].

This superior performance stems from CAP’s low-calcium hydration products (calcium alumino-phosphate hydrates), which minimize Ca(OH)_2_ formation and reduce carbonation driving force [42]. Unlike Portland-based systems that degrade via decalcification and pore expansion, CAP undergoes mild, controlled carbonation with aragonite formation that secondarily fills pores, reducing defect density from 0.14% to 0.07% (XCT results). This positive feedback between carbonation, filling, and densification is rarely reported for conventional cements, positioning CAP as a promising “system replacement” solution for extreme CCUS-EOR environments.

In summary, the two systems represent two effective strategies for improving the carbonation resistance of cement. The PANI@TiO_2_-modified system strengthens the cement matrix through external and active interface regulation, whereas the CAP system resists corrosion by virtue of its inherent chemical and structural stability. This study cross-validated the above mechanisms through multiple characterization methods, providing a solid theoretical basis for the design and selection of high-performance cement materials for CCUS-EOR environments.

## 5. Conclusions

This study systematically investigated the carbonation resistance and mechanism of PANI@TiO_2_-modified cement (P@T) and phosphoaluminate cement (CAP) under simulated CCUS-EOR conditions (130 °C, 25 MPa, 7 MPa CO_2_ partial pressure) and compared them with silica-fume-containing Class G oil-well cement (PT). Results show that carbonation resistance follows the order CAP > P@T > PT. CAP is inferred to exhibit a potential “corrosion-induced densification” behavior, with continuously increased compressive strength and decreased permeability over 90 days, owing to its low-calcium hydration products and secondary pore-filling by aragonite. P@T is suggested to achieve the possible decoupling of high carbonation degree and microstructural stability by regulating carbonate crystallization to refine and homogenize products, thereby maintaining structural integrity. In contrast, PT fails completely at 60 days. The P@T system relies on multi-scale active protection (physical filling, proton buffering, interface regulation), while CAP relies on intrinsic chemical inertness, representing two feasible technical paths: “matrix modification” and “cement system replacement”. In terms of engineering trade-offs, P@T offers good compatibility with existing field-mixing processes and lower cost, but its long-term performance is slightly inferior to CAP; CAP has ultra-high durability but higher material cost. Major challenges include the long-term stability of CAP beyond 90 days and the dispersion uniformity of PANI@TiO_2_ in large-scale slurry. Future work can focus on long-term corrosion tests lasting over 180 days, field-scale simulations, and economic optimization so as to promote the practical application of high-performance anti-carbonation cement systems in CCUS-EOR well engineering.

## Figures and Tables

**Table 1:** Chemical compositions of different types of oil-well cement (wt%).

Cement Type	CaO	SiO_2_	Fe_2_O_3_	Al_2_O_3_	MgO	TiO_2_	SO_3_	Others
Class G oil-well cement	64.91	22.80	4.37	2.82	1.34	-	1.94	1.80
CA50-grade aluminate cement	35.31	7.28	1.12	53.15	-	1.61	0.45	1.08

**Table 2:** Compositions of different types of oil-well cement slurry (wt%).

Sample	Class G Cement	CA50-Grade Aluminate Cement	Fresh Water	Retarder	Superplasticizer	Defoamer	Silica Fume	PANI@TiO_2_	Sodium Hexametaphosphate
PT	100	-	54	4	1	1	35	-	-
P@T	100	-	54	4	1	1	35	1	-
CAP	-	100	68	-	-	-	35	-	5

**Table 3:** Composition of simulated saline water (mg/L).

Ion Content							
HCO_3_^−^	CO_3_^2−^	Cl^−^	SO_4_^2−^	Ca^2+^	Mg^2+^	Na^+^	K^+^
3112.02	60.02	11,166.75	119.11	372.74	67.82	3523.4	4441.5

**Table 4:** XCT scanning parameters and standard settings.

Parameter	Standard Setting
X-ray tube voltage	120 kV
X-ray tube current	0.12 mA
Detector type	DXR.250
Rotation angle	360°
Detector unit	2014
Number of projections	1000
Number of pixels	2014 × 2014

**Table 5:** Detailed defect information of cement stones in different systems at different corrosion ages.

Sample	0 d			28 d		
	Material Volume (mm^3^)	Defect Volume (mm^3^)	Defect Volume Ratio (%)	Material Volume (mm^3^)	Defect Volume (mm^3^)	Defect Volume Ratio (%)
PT	7926.38	40.13	0.50	11,023.47	605.95	5.21
P@T	12,017.93	22.40	0.19	10,498.29	32.63	0.31
CAP	11,328.87	16.37	0.14	11,535.2	8	0.07

## Data Availability

The original contributions presented in this study are included in the article/Supplementary Materials. Further inquiries can be directed to the corresponding authors.

## Supplementary Materials

The following supporting information can be downloaded at: https://www.mdpi.com/article/10.3390/ma19112279/s1, Figure S1: Tukey multiple comparison results for compressive strength; Figure S2: Tukey multiple comparison results for permeability; Figure S3: Tukey multiple comparison results for porosity.
