# Supplementary material for: Research on Carbonation Resistance of Modified/Non-Portland Cements in Carbon Capture, Utilization, and Storage-Enhanced Oil Recovery
Source: Materials (Basel). 2026 May 28;19(11):2279. doi: 10.3390/ma19112279 (PMC13257833; doi:10.3390/ma19112279)
Supplement: Supplementary file 1 [file materials-19-02279-s001.zip › materials-4290982-supplementary.pdf]

Supplementary Materials

Yaqiong Cao[1,*], Rengguang Liu[1], Shiming Zhou[1,*], Qian Tao[1], and Luo Liu[1]

[1] SINOPEC Research institute of Petroleum Engineering Co., Ltd., 197 Baisha Road, Changping District, Beijing, 102206, China.

* Correspondence: caoyaqiong1992@163.com; Zhousm.sripe@sinopec.com.

Statistical Analysis

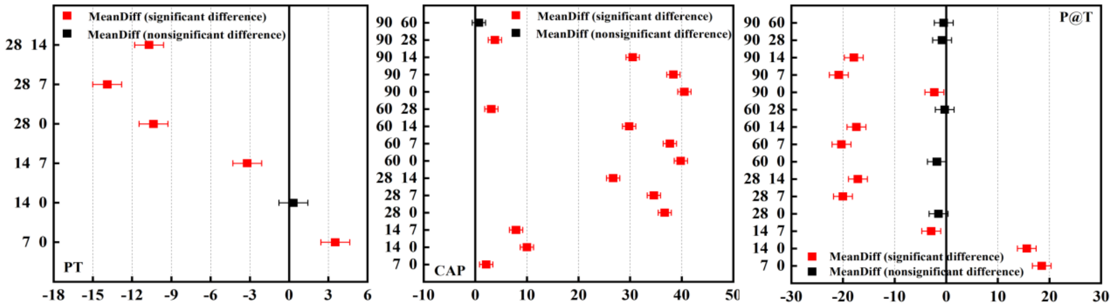

Figure S1. Tukey multiple comparison results for compressive strength

Figure S1 presents the Tukey multiple comparison results for compressive strength of the PT group, CAP group, and P@T group. Red squares indicate significant differences between groups ($p<0.05$), while black squares indicate non-significant differences ($p\geq0.05$). The X-axis represents the mean difference between two groups, and the Y-axis shows the paired age combinations (later age vs. earlier age). The analysis shows that for the PT group, the strength increased significantly by approximately 3 MPa at the early stage (0→7 d), but then decreased significantly by about 3 MPa at the middle stage (7→14 d), resulting in no significant difference between the 14 d strength and the initial value. At the later stage (14→28 d), all comparisons showed significant negative values (decreases of 10–15 MPa), indicating continuous deterioration and eventual structural damage. The CAP group exhibited an excellent increasing trend throughout the entire period. In the early stage (0→7 d), the strength increased significantly by about 5 MPa. From the middle to late stages (7→90 d), all comparisons between adjacent ages showed significant positive values. The 90 d strength was significantly higher than that at all earlier ages (increase of 184%, reaching 62.5 MPa), with only the 60→90 d comparison being non-significant, reflecting a slower growth rate at the later stage while still maintaining a high level, which verifies the potential corrosion-induced densification effect. The P@T group showed the largest early-stage increase (0→7 d: significant increase of about 18 MPa), followed by a significant decrease of about 5 MPa at the middle stage (7→14 d). In the later stage (14→90 d), the decreases were negative but not significant, and the 90 d strength remained significantly higher (about 10 MPa) than the initial value, indicating that PANI@TiO_2 modification effectively retarded the deterioration process, resulting in long-term performance superior to the initial state.

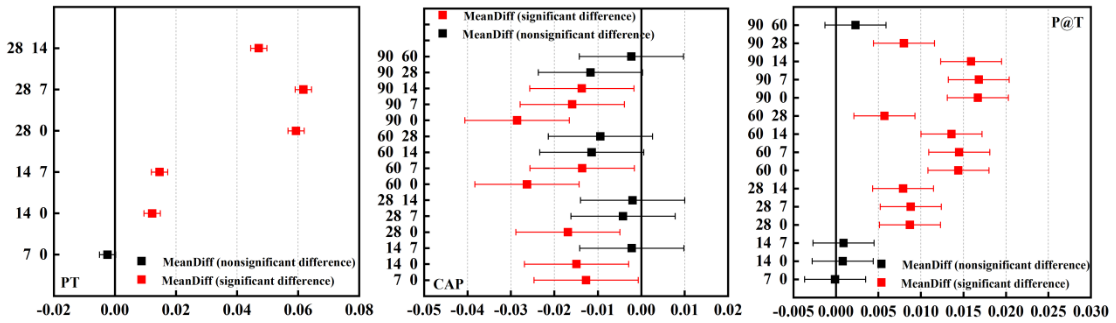

Figure S2. Tukey multiple comparison results for permeability

Tukey multiple comparison results further reveal the evolution of permeability for each group of cement stone (**Figure S2**). For the CAP group, comparisons with the initial value (0 d) from 7 d to 90 d (7 0, 14 0, 28 0, 60 0, 90 0) all showed significant negative differences (red squares), indicating that permeability decreased continuously and significantly with curing age. In particular, the significant negative difference for 90 0 directly validates that the permeability at 90 d decreased to 13.3% of its initial value (0.0044 mD), supporting the "corrosion-induced densification effect". Only the 90 60 comparison showed an error bar crossing the zero line (black square), indicating a slower rate of permeability decrease at the later stage (60→90 d), although the permeability remained at a very low level overall. For the P@T group, the mean differences in the early stage (0→7 d, 7→14 d) were all close to the zero line (black squares) and not significant, suggesting no obvious change in permeability during the initial period. In the later stage (14→28 d, 28→60 d, 60→90 d), all comparisons showed small positive values (some as red squares), reflecting a slow increase in permeability. Although the mean difference for 90 0 was positive, its magnitude was very small, confirming that "the modification effectively retarded structural deterioration, and the permeability at 90 d was only slightly higher than the initial value (0.0288 mD)", with the degree of deterioration being far lower than that of the PT group. For the PT group, the mean difference in the early stage (0→7 d) was close to the zero line (black square), corresponding to the hydration shrinkage phase where permeability showed no significant change. From the middle to the later stage (7→14 d, 14→28 d), the mean differences were all significantly positive (red squares), indicating a marked increase in permeability. Among these, the significant positive difference for 28 0 directly validates the "sharp increase in permeability by 269.86% to 0.0812 mD within 28 d", reflecting enhanced pore network connectivity and aggravated structural deterioration.

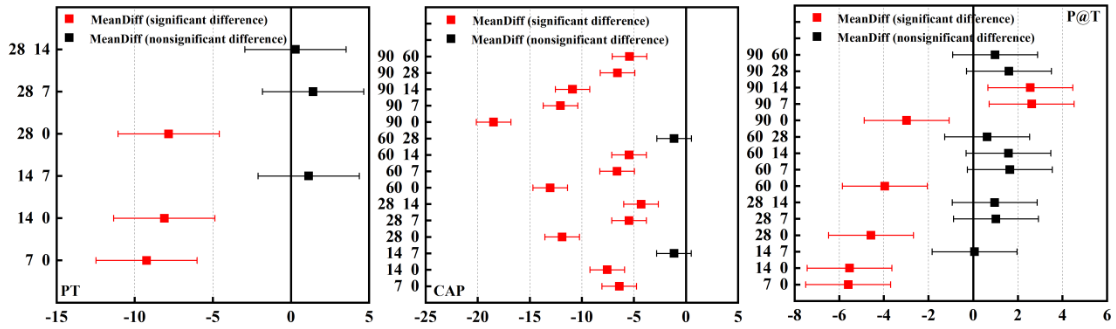

Figure S3. Tukey multiple comparison results for porosity

Tukey multiple comparison results further reveal the evolution of porosity for each group of cement stone (**Figure S3**). For the CAP group, comparisons with the initial value (0 d) from 7 d to 90 d (7 0, 14 0, 28 0, 60 0, 90 0) all showed significant negative differences (red squares),

indicating that porosity decreased continuously and significantly with curing age. In particular, the significant negative difference for 90 0 directly verifies that the porosity at 90 d decreased to approximately half of its initial value (37.44%→18.97%), supporting the micro-mechanism of "corrosion-induced densification". Only the 90 60 comparison showed an error bar crossing the zero line (black square), indicating a slower rate of porosity decrease at the later stage, with the densification process tending to stabilise. For the P@T group, the mean differences in the early stage (0→7 d, 7→14 d) were significantly negative (red squares), indicating a slight decrease in porosity during the early period, corresponding to the pore structure optimisation effect of the modifier. In the later stage (14→28 d, 28→60 d, 60→90 d), the mean differences were small positive values (some as black squares), indicating a slow recovery of porosity, but most of the differences were not significant, and the porosity was maintained in the range of 27.82%–30.44% overall. The mean difference for 90 0 was close to the zero line (black square), indicating that the porosity at 90 d was not significantly different from the initial value, confirming the effect of the modifier in effectively retarding structural deterioration. For the PT group, the mean differences in the early stage (0→7 d, 7→14 d) were significantly negative (red squares), reflecting a significant decrease in porosity during the early period, corresponding to the hydration shrinkage stage of cement. In the middle stage (14→28 d), the mean difference was significantly positive (red square), indicating a significant rebound in porosity, which verifies the description of porosity rebounding to 35.63% in the middle stage and reflects structural deterioration caused by CO_2 corrosion. It is worth noting that the mean difference for 28 0 was significantly negative (red square), indicating that although porosity rebounded in the middle stage, the porosity at 28 d was still significantly lower than the initial value (0 d), consistent with the trend of "first decreasing then increasing" without exceeding the initial level in the later stage.
